# PPARβ/δ Agonism Upregulates Forkhead Box A2 to Reduce Inflammation in C2C12 Myoblasts and in Skeletal Muscle

**DOI:** 10.3390/ijms21051747

**Published:** 2020-03-04

**Authors:** Wendy Wen Ting Phua, Wei Ren Tan, Yun Sheng Yip, Ivan Dongzheng Hew, Jonathan Wei Kiat Wee, Hong Sheng Cheng, Melvin Khee Shing Leow, Walter Wahli, Nguan Soon Tan

**Affiliations:** 1 School of Biological Sciences, Nanyang Technological University Singapore, 60 Nanyang Drive, Singapore 637551, Singapore; WPHUA003@e.ntu.edu.sg (W.W.T.P.); ysyip@ntu.edu.sg (Y.S.Y.); IHEW001@e.ntu.edu.sg (I.D.H.); wkwee@ntu.edu.sg (J.W.K.W.); hscheng@ntu.edu.sg (H.S.C.); 2 NTU Institute for Health Technologies, Interdisciplinary Graduate School, Nanyang Technological University Singapore, Singapore 637551, Singapore; 3 Lee Kong Chian School of Medicine, Nanyang Technological University Singapore, 11 Mandalay Road, Singapore 308232, Singapore; weiren.tan@ntu.edu.sg (W.R.T.); melvin_leow@ntu.edu.sg (M.K.S.L.); Walter.Wahli@unil.ch (W.W.); 4 Department of Endocrinology, Division of Medicine, Endocrine and Diabetes Clinic, Tan Tock Seng Hospital, 11 Jalan Tan Tock Seng, Singapore 308433, Singapore; 5 INRA ToxAlim, UMR1331, Chemin de Tournefeuille, Toulouse Cedex 3, 31300 Toulouse, France; 6 Center for Integrative Genomics, Université de Lausanne, Le Génopode, CH-1015 Lausanne, Switzerland

**Keywords:** peroxisome proliferator-activated receptors β/δ, Forkhead box A2, muscle inflammation, tetanic contraction

## Abstract

Daily activities expose muscles to innumerable impacts, causing accumulated tissue damage and inflammation that impairs muscle recovery and function, yet the mechanism modulating the inflammatory response in muscles remains unclear. Our study suggests that Forkhead box A2 (FoxA2), a pioneer transcription factor, has a predominant role in the inflammatory response during skeletal muscle injury. FoxA2 expression in skeletal muscle is upregulated by fatty acids and peroxisome proliferator-activated receptors (PPARs) but is refractory to insulin and glucocorticoids. Using PPARβ/δ agonist GW501516 upregulates FoxA2, which in turn, attenuates the production of proinflammatory cytokines and reduces the infiltration of CD45+ immune cells in two mouse models of muscle inflammation, systemic LPS and intramuscular injection of carrageenan, which mimic localized exercise-induced inflammation. This reduced local inflammatory response limits tissue damage and restores muscle tetanic contraction. In line with these results, a deficiency in either PPARβ/δ or FoxA2 diminishes the action of the PPARβ/δ agonist GW501516 to suppress an aggravated inflammatory response. Our study suggests that FoxA2 in skeletal muscle helps maintain homeostasis, acting as a gatekeeper to maintain key inflammation parameters at the desired level upon injury. Therefore, it is conceivable that certain myositis disorders or other forms of painful musculoskeletal diseases may benefit from approaches that increase FoxA2 activity in skeletal muscle.

## 1. Introduction

Skeletal muscle, which accounts for 40% to 50% of total body weight in a healthy individual, plays multiple bodily functions [1]. Its structure allows voluntary movement and posture maintenance through coordinated contraction and relaxation of muscle fibers, while withstanding massive and sudden mechanistic and bioenergetic changes [2]. Muscle injury can occur through diverse mechanisms such as mechanical injury, muscular dystrophies, infectious disease, and biochemical toxicities. Despite the type of injuries, the general injury and repair mechanism is similar. It consists of overlapping phases of degeneration, inflammation, regeneration, and fibrosis [3]. In the early phase of muscle injury, muscle-derived cytokines and chemokines, i.e., myokines, released by the injured muscle trigger an inflammatory cell invasion. Polymorphonuclear leukocytes first arrive to the site of injury, which are eventually replaced by monocytes. Over the next one to two days, monocytes differentiate into macrophages that phagocytose and remove necrotic tissue [4]. Macrophages, along with fibroblasts, produce myogenic factors to activate the regenerative mechanisms that involve satellite cells [5,6]. Various studies have contributed to a complex picture in which inflammation promotes both injury and repair. Whether the inflammatory process has an overall beneficial or detrimental effect on muscle function is influenced by the magnitude of the response, among others [7]. A dysregulation of the early myokine production has a cascading impact on the repair process. However, the mechanism that modulates the extent and magnitude of inflammatory response remains unclear. 

Transcriptional control plays an important role in regulating the responses of the skeletal muscle to diverse stimuli [8]. Some of them are mediated by peroxisome proliferator-activated receptors (PPARs) [9]. The three PPAR isotypes, designated PPARα, PPARβ/δ, and PPARγ, belong to the nuclear hormone receptor superfamily. They are activated by endogenous ligands, such as free fatty acids and their derivatives, and by a variety of synthetic drugs used in the treatment of metabolic syndrome and type 2 diabetes [10,11]. PPARβ/δ is the predominant isotype in skeletal muscle and its activation increases lipid uptake and catabolism via β-oxidation [12], along with a shift towards oxidative fibers, which enhances oxidative capacity and promotes running endurance, leading to an overall reduction in body fat [13,14,15]. Conversely, selective PPARβ/δ ablation in skeletal muscle leads to lower oxidative capacity in the fibers, resulting in obesity and diabetes [16]. The regulatory roles of PPARβ/δ in the lipid metabolism, metabolic reprogramming, and mitochondrial activity of skeletal muscles can also play a role in the protection against insulin resistance and type 2 diabetes [17,18]. While the role of PPARβ/δ in skeletal muscle metabolism is already well studied, its function in muscle inflammation and damage is unclear. 

Pioneer factors are transcription factors that can directly bind condensed chromatin. They can increase or decrease gene transcription by establishing the competency for gene expression. Transcription factor Forkhead box A2 (FoxA2) is a canonical example of a pioneer factor that influences the transcriptional activity of many nuclear hormone receptors and other genes [19]. FoxA2 orchestrates the transcriptional regulation of glucose and lipid homeostasis in metabolically active tissues such as liver, pancreatic β-cells, and adipocytes [20,21,22]. Most studies on FoxA2 investigated its role in the liver, where it executes the liver gluconeogenic program by integrating the transcriptional responses of hepatocytes to hormonal stimulation [21,23]. FoxA2 cooperates with PPARα, PPARγ, and liver X receptor in aged and fatty liver to regulate the expression of target genes [24]. In the epididymis, but not prostate, FoxA2 colocalizes with the androgen receptor in the promoter of epididymis-specific genes and promotes their regulation [25]. The role of FoxA2 in skeletal muscle and its functional interactions with PPARβ/δ, if any, are unknown. 

This study revealed a hitherto unexpected function of FoxA2 in skeletal muscle. We found that ligand-activated PPARβ/δ enhanced FoxA2 expression in C2C12 myoblasts and in skeletal muscle. In vivo, PPARβ/δ-mediated upregulation of FoxA2 in the skeletal muscle reduced local muscle inflammation and improved muscle force generation. This PPARβ/δ-FoxA2 axis represents a potential novel therapeutic target to improve muscle health. It is conceivable that certain myositis disorders or other forms of painful musculoskeletal diseases could benefit from approaches that increase FoxA2 activity in skeletal muscles.

## 2. Results

### 2.1. FoxA2 Regulates Genes Associated with Inflammatory Response to Injury

**Figure 1:**
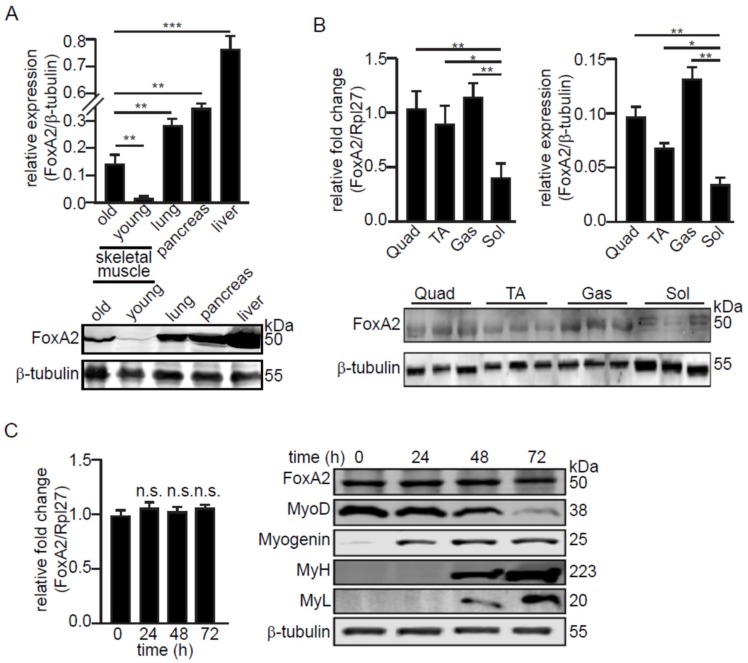
FoxA2 is expressed in C2C12 myoblasts and in skeletal muscle. (**A**) Relative FoxA2 expression in various mouse tissues; (**B**) in various hindlimb skeletal muscles; (**C**) and during myogenesis of C2C12 cells. Representative immunoblots of FoxA2, MyoD, myogenin, myosin heavy chain (MyH), and myosin light chain (MyL) are shown. β-tubulin serves as a loading control was from the same samples. Values are mean + SD from 3 independent experiments. * *p* < 0.05; ** *p* < 0.01; *** *p* < 0.0001; and n.s., not significant.

We first compared the relative expression of FoxA2 protein in the skeletal muscle, liver, lung, and pancreas (Figure 1A). FoxA2 was previously shown to be expressed in liver, lung, and pancreas [24,26]. The expression of FoxA2 in skeletal muscle was detected at a lower level than lung, pancreas, and liver. Skeletal muscle from five-month-old mice expressed a higher level of FoxA2 as compared with four-week-old mice (Figure 1A). Further analysis revealed that FoxA2 expression was higher in muscles with a larger proportion of type II fast-twitch fibers, such as gastrocnemius, quadriceps, and tibialis anterior (TA) as compared with type I slow-twitch fibers, such as soleus (Figure 1B). FoxA2 was also expressed in the C2C12 myoblast cell line. Its expression was unchanged during the differentiation of the myoblasts to myotubes, suggesting that FoxA2 is not involved in myoblast differentiation. Differentiation was confirmed by the expression of specific muscle proteins (MyoD, Myogenin, MyL, and MyH) (Figure 1C).

**Figure 2:**
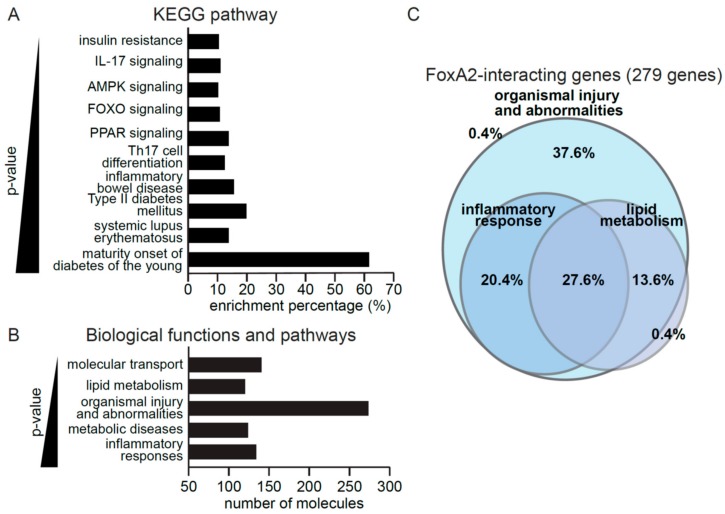
FoxA2 regulates genes associated with inflammation response to injury. (**A**,**B**) KEGG pathways (**A**) and top gene ontologies for biological functions (**B**) of FoxA2-regulated genes. A lower *p*-value indicates a stronger association with the indicated gene ontologies; (**C**) FoxA2 regulated genes play roles in various biological functions. A total of 279 genes are involved in metabolism, inflammatory response, and organismal injury. The percentage of genes implicated in the indicated biological functions is shown.

To gain insights into the biological processes controlled by FoxA2, downstream targets of FoxA2 from the Ingenuity Pathway Analysis data repository were analyzed, of which, a total of 279 target genes were identified. These genes were involved in inflammation, such as IL-17 signaling and inflammatory bowel disease, or energy homeostasis, such as insulin resistance (Figure 2A). PPAR and AMPK signaling pathways are associated with both inflammation and metabolism. Gene ontology analysis suggested a biological role of FoxA2 in inflammatory response, metabolic disease, organismal injury and abnormalities, lipid metabolism, and molecular transport (Figure 2B). Further interrogation revealed that 37.6% and 20.4% of the FoxA2 target genes were associated with organismal injury and abnormalities, and inflammatory response, respectively. Approximately 27.6% have overlapping functions in lipid metabolism, and inflammatory responses (Figure 2C). Our analyses suggested a role for FoxA2 in the regulation of inflammation response to injury.

### 2.2. PPARβ/δ Upregulates FoxA2 Expression in C2C12 Cells

FoxA2 expression is modulated by insulin and glucocorticoids in the liver [27]. These hormones also alter skeletal muscle metabolism and functions [28,29]. Thus, we examined if FoxA2 expression in C2C12 myotubes was similarly regulated through hormonal stimulation. The mRNA and protein levels of FoxA2 were not altered by hydrocortisone (cort), dexamethasone (Dex), or insulin (Figure S1A,B). The phosphoactivation of Akt1 by insulin was used as a positive control of hormone action. Low concentrations of cort and Dex were used to avoid the induction of the muscle atrophy-related genes (Atrogin and MuRF1) and the muscle negative regulatory factor myostatin (Figure S1B). The increased expression of Pdk4 by Dex and cort served as positive controls. As fatty acids have emerged as important signalling molecules during metabolism in diverse organs [30], we examined the effect of palmitic acid (PA) on FoxA2 expression. Real-time qPCR and Western blot analyses showed that PA increased FoxA2 expression in a dose-dependent manner (Figure S1C). Together, these results suggested a tissue-specific regulation of FoxA2 in C2C12 myoblasts.

**Figure 3:**
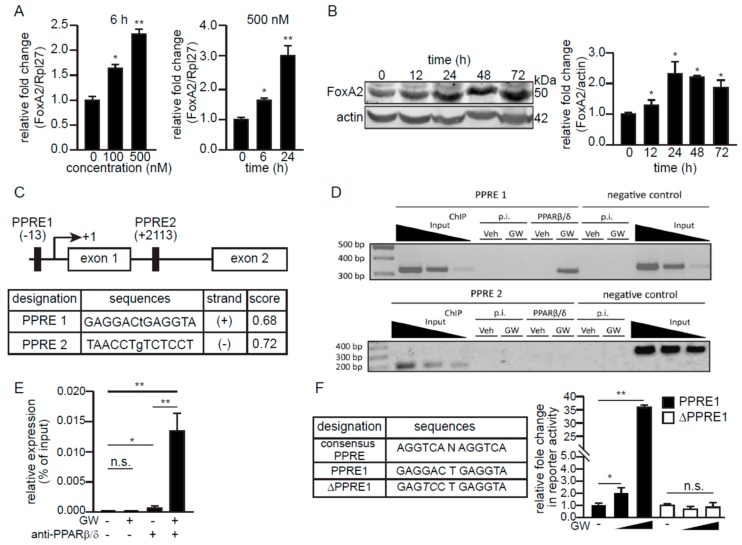
FoxA2 is a PPARβ/δ target gene in skeletal muscle. (**A**,**B**) Relative FoxA2 mRNA (**A**) and protein expression (**B**) in myotubes treated for the indicated time with indicated concentrations of GW501516 (GW). Representative immunoblots of FoxA2 are shown. Actin serves as a loading control from the same samples. Values are the mean + SD from 3 independent experiments. * *p* < 0.05; ** *p* < 0.01; and n.s., not significant; (**C**) A schematic diagram of putative PPREs in the promoter and the first intronic region of mouse *FoxA2* gene. The below table shows the putative PPRE sequences and scores, with a maximum score of 1.0, which reflect the predicted confidence using NUBIScan, a prediction software for nuclear receptor response elements; (**D**,**E**) Chromatin immunoprecipitation (ChIP) of predicted PPREs in *FoxA2* gene with PPARβ/δ antibody or pre-immune IgG (p.i.) in C2C12 treated with either vehicle (Veh) or GW (**D**). Quantitative ChIP-qPCR analysis of PPARβ/δ binding at PPRE1 in the presence or absence of GW treatment (**E**); (**F**) Relative luciferase activity from reporter gene constructs containing either PPRE1 or ΔPPRE1 in HEK293T cells in the presence or absence of 1 and 10 µM GW for 6 h. DMSO was used as the vehicle control. Values are the mean + SD from 3 independent experiments. * *p* < 0.05; ** *p* < 0.01; and n.s., not significant.

Since fatty acids and their derivatives are agonists of PPARs [31], we asked if FoxA2 expression could be regulated by PPARβ/δ, the predominant PPAR isotype in skeletal muscle [13]. GW501516 (GW), a highly selective PPARβ/δ agonist, increased the mRNA and protein levels of FoxA2 in C2C12 myotubes in a dose- and time-dependent manner (Figure 3A,B). In silico analysis of the regulatory region at the promoter and the first intron of the mouse *FoxA2* revealed two putative PPAR response elements (PPRE1 and PPRE2) (Figure 3C). Chromatin immunoprecipitation (ChIP) analysis of GW-treated C2C12 myoblasts revealed the binding of PPARβ/δ to PPRE1, but not to PPRE2 of *FoxA2* (Figure 3D). The interaction of PPARβ/δ with the *FoxA2* promoter region was also validated by ChIP-qPCR. There was some PPARβ/δ interaction with PPRE1 at the basal level, and this occupancy was increased by GW treatment of the cells (Figure 3E). Finally, reporter luciferase activities were increased by GW in HEK293 cells transfected with the PPRE1 reporter vector, but not with the vector containing ΔPPRE1 (Figure 3F). The ΔPPRE1 was mutated at the +4 and +5 positions of PPRE1 sequence. In summary, we identified mouse *FoxA2* as a direct target gene of PPARβ/δ in C2C12 myoblasts.

### 2.3. Ligand-Activated PPARβ/δ Upregulates FoxA2 to Attenuate LPS-Induced Inflammation

**Figure 4:**
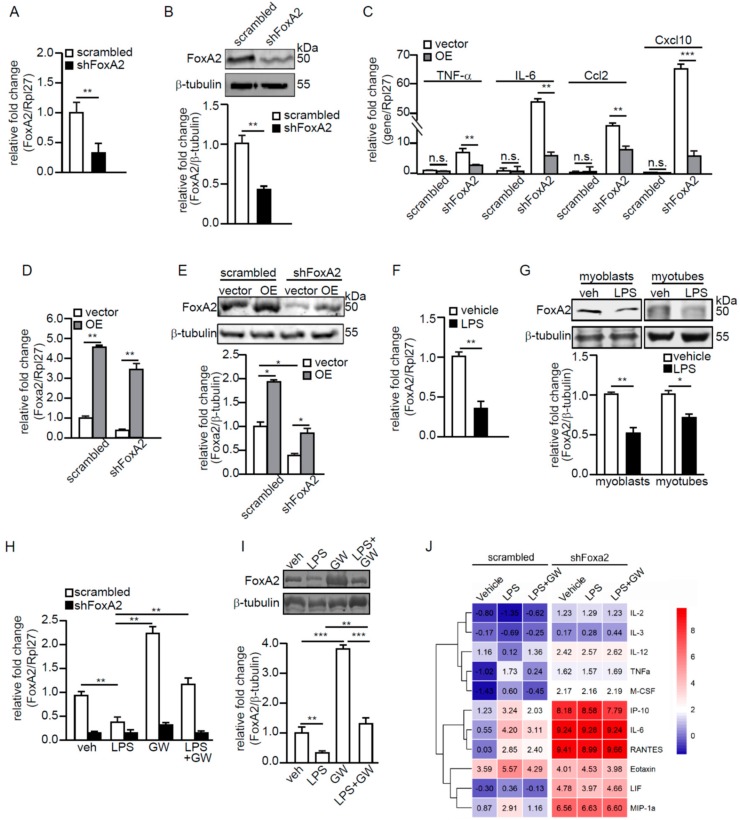
FoxA2 attenuates LPS-induced inflammation in C2C12 myoblasts. (**A**,**B**) Relative fold change in FoxA2 mRNA (**A**) and protein (**B**) levels in scrambled or shFoxa2-knockdown C2C12 cells; (**C**) Relative fold change in mRNA levels of selected proinflammatory genes in shFoxA2-knockdown and scrambled C2C12 cells transfected with empty vector (vehicle) or expression vector containing FoxA2 (OE) cDNA; (**D**–**I**) Relative fold change in FoxA2 mRNA (**D**,**F**,**H**) and protein (**E**,**G**,**I**) levels in shFoxA2-knockdown, scrambled, and FoxA2-overexpressing C2C12 cells (**D**,**E**), in LPS- (100 ng/mL) or vehicle-treated C2C12 cells (**F**,**G**) and in the presence or absence of GW501516 (GW) (**H**,**I**); (**J**) Cytokine concentrations in conditioned medium of scrambled and shFoxA2-knockdown cells subjected to indicated treatments for 24 h. Heatmap shows the log_2_ value of the concentrations. Representative immunoblots of FoxA2 are shown. Β-tubulin serves as a loading control is from the same samples. Values are the mean + SD from 3 independent experiments. * *p* < 0.05; ** *p* < 0.01; *** *p* < 0.0001; and n.s., not significant.

Next, the effect of reduced FoxA2, through shRNA inhibition, in C2C12 myoblasts (shFoxA2) was studied. The FoxA2 level was reduced by ~70% as confirmed by RT-qPCR and immunoblot analyses (Figure 4A,B). Significant increases in the mRNA levels of proinflammatory cytokines and chemokines, such as IL-6, TNF-α, Ccl2, and Cxcl10 were also detected in shFoxA2-C2C12 myoblasts (Figure 4C). Next, we overexpressed FoxA2 in shFoxA2- and scrambled-C2C12 myoblasts via the transfection of a mammalian expression vector containing FoxA2 cDNA. The expressions of proinflammatory cytokines and chemokines were significantly reduced by FoxA2 overexpression in shFoxA2-C2C12 as compared with empty vector (Figure 4C). The mRNA and protein levels of FoxA2 were increased by approximately four-fold and approximately two-fold in these transfected cells, respectively (Figure 4D,E).

A reduced FoxA2 expression in C2C12 myoblasts resulted in an exacerbated inflammatory response. We hypothesized that FoxA2 attenuates inflammation to protect against inflammation-associated muscle injury. Lipopolysaccharide (LPS) is a classic activator of inflammation. FoxA2 expression was reduced by 58% in C2C12 myoblasts treated with 100 ng/mL LPS as compared with the vehicle-treated cells (Figure 4F). FoxA2 protein was similarly reduced by 30% in myotubes treated with LPS (Figure 4G). Subsequently, scrambled- and shFoxA2-C2C12 myoblasts were treated with LPS in the presence or absence of GW for 24 h. The expression of FoxA2 was partially rescued when cells were cotreated with LPS and GW (Figure 4H,I). The shFoxA2-C2C12 cells were not significantly affected by either treatment (Figure 4H). LPS alone caused an increase in inflammatory cytokines and chemokines in the conditioned medium of C2C12 myoblasts as compared with vehicle-treated cells. We observed a reduction in cytokine concentrations in the conditioned medium of C2C12 myoblasts cotreated with LPS and GW (Figure 4J). An increased expression of inflammatory cytokines was detected in shFoxA2-C2C12 myoblasts as compared with scrambled control cells. The cotreatment of LPS and GW did not attenuate the inflammatory status shFoxA2-C2C12 cells, most likely due to the already exacerbated cytokine production. Overall, these findings suggest that FoxA2 modulates the inflammatory response in skeletal muscles.

### 2.4. Skeletal Muscle FoxA2 Diminishes Inflammation-Associated Tissue Damage

The administration of LPS in mice is an established model that reproducibly induces skeletal muscle inflammation without injury [32,33]. A comparison with saline-treated mice showed that LPS increased the mRNA expression of IL-6, TNF-α, Ccl2, and Cxcl10 by more than four-fold, while pretreatment with GW effectively attenuated the LPS-induced overexpression of these inflammatory genes (Figure S2A). Pretreatment with GW also normalized the expression of FoxA2 in gastrocnemius muscle, which was otherwise suppressed by LPS (Figure S2B). However, the systemic nature of the LPS-mediated inflammatory response limits its usefulness in examining the effects of local muscle inflammation on contractile function.

**Figure 5:**
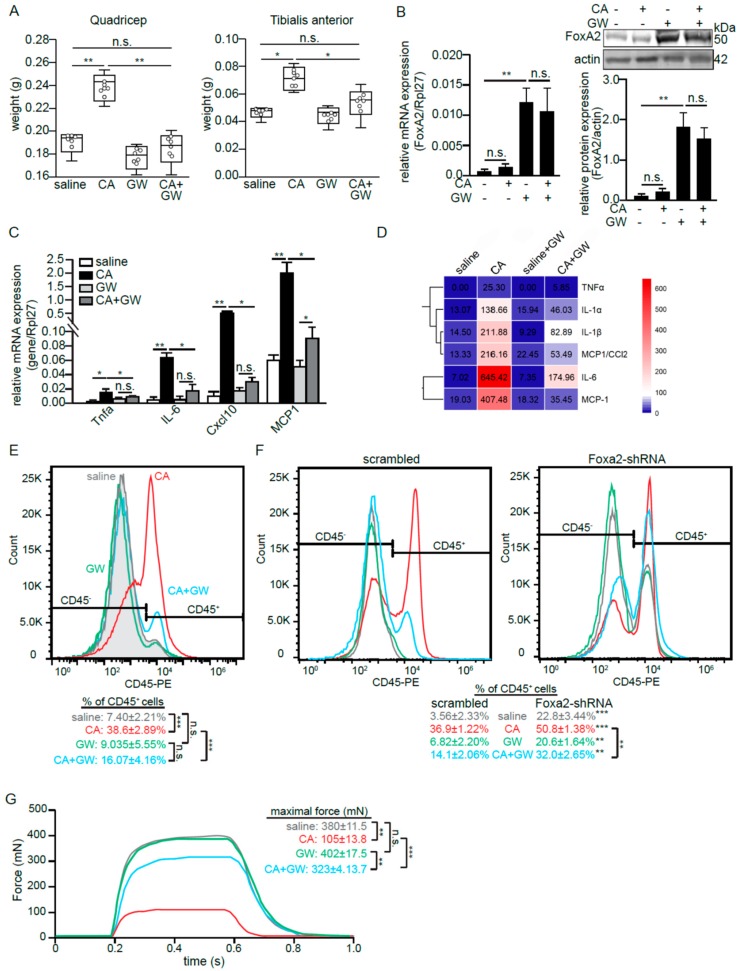
FoxA2 diminishes inflammation-associated muscle damage. (**A**) Mean weight of quadricep and tibialis anterior (TA) muscles at 6 h after saline or CA treatment on contralateral muscles. Hollow circles in the box-and-whiskers boxes represent the individual data points; (**B**–**D**) Relative FoxA2 (**B**) and pro-inflammatory genes (**C**,**D**) mRNA (B left panel, C), protein (B right panel, D) expression in TA muscles at 6 h of CA-induced inflammation in the presence or absence of GW501516 (GW). Heatmap showing mean cytokine concentrations from isolated TA muscles; (**E**,**F**) Representative histogram plots of infiltrated CD45-positive (CD45+) immune cells from TA muscle from wildtype mice (**E**), as well as muscle Ad-shFoxA2 and Ad-scrambled mice (**F**) subjected to indicated treatments; (**G**) Mechanical properties of EDL muscle from wild-type mice subjected to indicated treatments. Representative plots show maximal tetanus force recorded. Five mice per experiments are used for each treatment. *n* = 3 independent studies. * *p* < 0.05; ** *p* < 0.01, *** *p* < 0.001; and n.s., not significant.

An alternative, intramuscular injection of carrageenan (CA) is a well-established intervention for inducing acute and localized skeletal muscle inflammation in vivo [34]. It mitigates the confounding effects of the direct mechanical damage that occurs to muscle tissue during injury. CA triggered a local inflammation and edema as reflected by an increase in the weights of the quadricep and TA muscles (Figure 5A). CA alone did not alter FoxA2 expression in the tissues (Figure 5B). We also detected a higher expression of several proinflammatory cytokines and chemokines (Figure 5C,D), and greater infiltration of CD45+ immune cells (Figure 5E) in CA-treated as compared with saline-treated muscles. The cross-sectional H&E stains of TA muscles showed tissue damages and a huge infiltration of mononucleated cells into CA-induced tissue as compared with saline-treated tissue (Figure S2C). GW increased FoxA2 expression in TA muscles and did not impact the basal local cytokine expression as compared with saline (Figure 5C–E). The intramuscular co-injection of GW and CA (CA + GW) elevated FoxA2 expression. Importantly, in this co-injection condition, GW significantly reduced the inflammatory response with less tissue damage as compared with CA injection alone (Figure 5 and Figure S2A). To confirm that the effect of GW was mediated via PPARβ/δ, similar experiments were performed on PPARβ/δ-deficient mice. The PPARβ/δ deficiency significantly diminished the effect of GW on immune cell infiltration (Figure S2D). 

FoxA2 deficiency in mouse results in embryonic lethality and a muscle-specific FoxA2 deleted mouse is not available. We circumvented these limitations using adenovirus (Ad)-mediated shRNA knockdown of FoxA2 in skeletal muscle. The in vivo FoxA2 protein in the skeletal muscle was reduced by ~75% as compared with collateral scrambled shRNA treated muscle (Figure S2E). The reduction in FoxA2 resulted in a higher infiltration of CD45+ cells into the skeletal muscle even in the saline control, which was further exacerbated by CA treatment as compared with Ad-scrambled shRNA treatment (Figure 5F). In the CA + GW cotreatment, the anti-inflammatory effect of GW was significantly diminished in FoxA2 Ad-shRNA treatment as evidenced by a small reduction in the percentage of infiltrating CD45+ immune cells as compared with Ad-scrambled shRNA treatment (Figure 5F). 

Skeletal muscle plays a pivotal role in force generation for locomotion. To understand the impact of this inflammation-associated damage on force generation, we measured the maximal tetanic contraction of similarly treated EDL muscles upon direct electrical stimulation. Consistent with the above observation, CA severely diminished the tetanic contraction as compared with the saline-treated muscle, but GW largely attenuated this effect (Figure 5G). Taken together, we showed that PPARβ/δ-mediated upregulation of FoxA2 attenuates the infiltration of immune cells and reduces inflammation-associated tissue damage.

## 3. Discussion

Our study suggests that FoxA2 in skeletal muscle has a predominant role in the inflammatory response to injury. The expression of FoxA2 in skeletal muscle was upregulated by fatty acids and PPARβ/δ agonist, but not by insulin and glucocorticoids. We show that PPARβ/δ-mediated upregulation of FoxA2 attenuates the production of proinflammatory myokines and reduces the infiltration of CD45+ immune cells. Consequently, the reduced local inflammation limits tissue damage and promotes the restoration of muscle function.

Adult skeletal muscle is subjected to a continuous and frequent turnover, not only in response to a prominent injury or repetitive traumas. Muscle damage also occurs during prolonged or intense exercise, such as a marathon [35]. Exercise-induced myokines often provide beneficiary effects on muscle regeneration, counteract systemic inflammation, and modulate glucose and lipid metabolism [36]. However, very high-intensity exercise bouts are known to trigger systemic inflammation, a subsequent immunodepression, and thus a higher risk for infections [37]. Excessive inflammation potentially leads to detrimental effects of secondary damage, increased muscle catabolism, and the development of muscle fibrosis [38,39]. This balance of pro- and anti-inflammatory cytokines is crucial for the coordination of muscle repair and regeneration. The knockdown of FoxA2 in C2C12 myoblast and skeletal muscle resulted in a tremendous increase in inflammatory myokines, an exacerbated infiltration of immune cells, and collateral tissue damage. PPAR β/δ-mediated upregulation of FoxA2 mitigated the inflammation-associated muscle damage. For example, we observed an increase in IL-6 expression in LPS-treated C2C12, which was attenuated by cotreatment with PPARβ/δ agonist GW. Similarly, CA-treated muscle caused an approximately 90- and approximately five-fold increase in IL-6 production and infiltrating CD45+ cells, respectively as compared with the saline treatment. The GW cotreatment led to a ~73% and ~58% decrease in IL-6 production and infiltrating CD45+ cells as compared with the CA treatment alone. FoxA2 has been shown to negatively regulate IL-6 production [40]. While IL6-mediated signaling has been associated with myogenesis and beneficial regulation of energy metabolism after exercise, it has long-term implications for persistent inflammatory conditions and chronic diseases. The regulatory role of FoxA2 in skeletal muscle was reminiscent of that in the lungs. Tissue-specific deletion of FoxA2 in respiratory epithelial cells aggravated the expression and production of cytokines and chemokines, leading to spontaneous pulmonary inflammation and goblet cell metaplasia [41]. Muscle-specific deletion of FoxA2 is currently not available. Our Ad-mediated knockdown of FoxA2 in skeletal muscle resulted in a higher basal infiltration of CD45+ that was further exacerbated by CA treatment as compared with Ad-scrambled shRNA treatment. We have used two different muscle inflammation models and assessed the muscle performance in vitro to confirm a role for FoxA2 in muscle inflammation. A limitation of our study is the lack of muscle-specific FoxA2-deficient mice, which would allow us to evaluate in vivo muscle performance by treadmill fatigue test and whole-limb grip strength assay, among others. Nevertheless, our study reveals a hitherto unknown role for FoxA2 in skeletal muscle, helping maintain homeostasis, and acting as a gatekeeper to maintain key inflammation parameters at the desired level in the face of external and internal perturbations. Our data suggest that therapies that promote PPAR-mediated FoxA2 expression potentially curtail muscular inflammation to reduce secondary tissue damages. 

We observed that the FoxA2 expression was higher in skeletal muscle from five-month-old mice as compared with four-week-old mice. While the reason for this increase remains elusive, it could be associated with postnatal muscle fiber growth. It was reported that there is significant myofibre hypertrophy from three weeks of postnatal growth to adulthood, when the number of both myonuclei and satellite cells is already established by P21 in mouse [42]. We showed that FoxA2 expression did not increase in myoblast differentiation to myotubes. We alluded to a causal relationship between PPARβ/δ and interleukin-15 (IL-15). In vitro, IL-15 has been shown to induce skeletal muscle hypertrophy [43] and that it mediates mitochondrial activity through a PPARβ/δ dependent mechanism in skeletal muscle cells [44]. However, the in vivo role of IL-15 signaling in skeletal muscle physiology remains controversial. IL-15 transgenic mice were reported to run twice as long as littermate control mice, associated with high expression of intracellular mediators of oxidative metabolism, including PPARβ/δ [45]. However, mice deficient in the IL-15 receptor α also ran greater distances and displayed increasing fatigue resistance and exercise capacity [46]. Nonetheless, these phenotypes were similar to the activated PPARβ/δ transgenic mice [14,15]. Future studies should investigate the relationship among IL-15, PPARβ/δ, and FoxA2.

Daily activities expose muscles to numerous challenges that can lead to tissue damage, thus, the absence of a protective mechanism would probably be deleterious enough to result in early mortality. Therefore, repetitive mechanical contraction-induced inflammation is mitigated by PPARβ/δ and FoxA2. Physical stress is also typically accompanied by the elevation of cortisol in the plasma. It is conceivable that the skeletal muscle FoxA2, which does not respond to cortisol, may offer adaptive benefits [35]. Thus, FoxA2 most likely ensures that the organism’s daily activities do not trigger rhabdomyolysis. Of course, prolonged and severe muscular exertion can conceivably lead to much more intense tissue damage that outstrips the capacity of FoxA2 to cope and contain inflammation and tissue damage. Our finding suggests that PPARβ/δ upregulation of FoxA2 mitigates the detrimental effect of inflammation on muscle performance. The anti-inflammatory activity of PPARβ/δ in skeletal muscles has been reported in a recent study and is linked to improve insulin sensitivity [47]. Indeed, many proinflammatory cytokines such as IL-6, TNFα, and IL-1β, which are consistently overexpressed in our LPS and CA-induced inflammatory models, are insulin antagonistic. Such anti-inflammatory activity, along with PPARβ/δ-dependent fatty acid oxidation, could contribute to the ameliorative effects on obesity and type 2 diabetes [48].

It is tempting to speculate that certain autoimmune myositis disorders such as polymyositis or dermatomyositis, or other forms of musculoskeletal painful diseases could one day be mitigated or treated via an approach that upregulates FoxA2 activity in the muscles. PPARs, as ligand activated nuclear receptors, play crucial biological roles in many health conditions, including metabolic diseases and autoimmune diseases [11]. The use of specific PPARβ/δ agonists in humans could be beneficial to lipid oxidation and could even have an additional benefit by reducing inflammation and painful muscles in these rheumatology conditions. Indeed, the therapeutic effect of HPP593, a PPARβ/δ agonist, is tested in sporadic inclusion body myositis (NCT01524406). Although the outcome from the clinical trial is not yet available, the findings reveal the safety and clinical efficacy of PPARβ/δ agonist in muscular disorders.

## 4. Materials and Methods

### 4.1. Cell Culture

C2C12 myoblasts (ATCC, Manassas, VA, USA) were maintained in DMEM supplemented with 10% fetal bovine serum at 37 °C under a 5% CO_2_ atmosphere. The differentiation of myoblasts into myotubes was performed as previously described [49]. All cell culture treatments were performed in fresh serum-free medium.

### 4.2. Bioinformatics Analysis

Downstream gene targets of FoxA2 were identified using the Ingenuity Pathway Analysis data repository (Qiagen Inc, Hilden, Germany). The target genes were analyzed using core analysis to explore the enriched gene ontology and canonical pathways. KEGG pathway analysis was performed using Goseq package (version 1.34.1) in R.

### 4.3. Transient Transfection

The C2C12 myoblasts were transfected with plasmid harboring shRNA against FoxA2 using FuGene HD transfection reagent according to manufacturer’s recommendation (Promega, Madison, WI, USA). Transfected cells were incubated for 24 to 48 h prior to analysis. The sequences of shRNA are in Table S1.

### 4.4. Real-Time PCR (qPCR) and Immunoblot Analyses

The real-time qPCR and immunoblot analyses were performed as previously described [50,51]. The sequences of primers for qPCR are in Table S2. Antibodies used in this study included: p-Akt (Ser 473) (sc-514032), Akt (sc-81434), PPARβ/δ (sc-74517), and actin (sc-32251) from Santa-Cruz Biotechnology (Dallas, TX. USA); FoxA2 (D56D6) from Cell Signaling Technology (Danvers, MA, USA); MyH (N3.36), MyL (T14), and β-tubulin (E7) from Developmental Studies Hybridoma Bank (Iowa City, IA, USA); and IRDye^®^ secondary antibodies from LI-COR Biosciences (Lincoln, NE, USA). N3.36, T14, and E7 were deposited to the Developmental Studies Hybridoma Bank (Iowa City, IA, USA) by Blau, H.M. Stockdale, F.E. and by Klymkowsky, M., respectively.

### 4.5. Chromatin Immunoprecipitation (ChIP)

ChIP assay was performed as previously described [50,51]. The sequences of chIP primers are in Table S3.

### 4.6. Luciferase Reporter Assay 

Firefly and *Renilla* luciferase activities were determined using the Dual-Luciferase^®^ Reporter Assay System as described previously [52]. The pGL2-PPRE1-luc and pGL2-ΔPPRE-luc reporter plasmids were used (Table S4). After 16 h of post-transfection, the cells were incubated with indicated GW501516 concentrations for 6 h. DMSO was used as a vehicle control. 

### 4.7. Adenovirus-Mediated Intramuscular Injection

Adenovirus containing shRNA for mouse FoxA2 (Ad-mFoxA2) and scrambled shRNA (Ad-scrambled) were constructed using a BLOCK-iT Adenoviral RNAi Expression System accordingly to the manufacturer’s recommendation (Thermo Fisher Scientific, Waltham, MA, USA). A total of 2 × 10^10^ plaque forming units of adenovirus was injected intramuscularly into the fibers in the adult (6 weeks of age) EDL muscle [53]. Ad-mFoxA2 was injected into EDL muscle, while the collateral limb muscle was injected with Ad-scrambled. After 10 days post injection, the EDL muscle was isolated and immunoblot analysis was performed to examine the knockdown efficiency.

### 4.8. Animal Experiment 

The C57BL/6J male mice were housed in the specific pathogen-free facility with 12 h/12 h light/dark cycle with ad libitum access to chow diet and water. All experiments were performed on animals of 4 months of age. In the LPS-induced inflammatory model, muscle inflammation induced was performed a described previously [54], except that 15 µg/kg of GW501516 was injected subcutaneously 2 h and 24 h prior LPS injection. In the carrageenan-induced inflammatory model, carrageenan solution (3%) at 10 mg/kg body weight was injected into the hindlimb muscle. Cotreatment of GW501516 and carrageenan were also conducted at the same concentrations as above. Sterile saline solution was injected into the contralateral muscle which served as a control. All procedures were performed according to the University’s Institutional Animal Care and Use Committee guidelines (A0321, A0324). 

### 4.9. In Vitro Muscle Strength Assessment

EDL muscles were dissected and mounted into an in vitro muscle testing system (1200A, Aurora Scientific Inc, Ontario, Canada). The muscles were housed in a vertical organ bath containing Ringer solution and bubbled with 5% CO_2_ in O_2_ at 25 °C. The muscle was stimulated by a 701B stimulator delivering square wave 15 V pulses for 0.3 ms by means of 2 platinum electrodes running parallel on either side of the suspended muscle. Specific force was normalized to the muscle cross-sectional area (CSA = wet weight (mg)/length (mm) × 1.06 (density mg/mm^3^)). 

### 4.10. Histological Staining

Muscle tissues were fixed, embedded, and sectioned as previously described [55,56]. Stained tissue sections were imaged using slide scanner microscope Zeiss Axio Scan Z1 (Carl Zeiss, Oberkochen, Germany).

### 4.11. Cytokine Detection

Muscle tissues were dissected and lysed in mammalian protein extraction reagent (Thermo Fisher Scientific, Waltham, MA, USA) supplemented with complete protease inhibitor mix (Roche Applied Science, Penzberg, Germany). Multiplex immunoassays were measured using BioPlex 200 system (Bio-Rad Laboratories Inc., Hercules, CA, USA) by Eve Technologies, Canada.

### 4.12. Statistical Analysis

Statistical analyses were performed using either two-tailed student’s t-test for group treatment in C2C12 myoblasts or two-tailed non-parametric Mann–Whitney test for 2 variables. *p*-value < 0.05 is considered significant.

## Supplementary Materials

Supplementary Materials can be found at https://www.mdpi.com/1422-0067/21/5/1747/s1.
