# Supplementary material for: PPARβ/δ Agonism Upregulates Forkhead Box A2 to Reduce Inflammation in C2C12 Myoblasts and in Skeletal Muscle
Source: Int J Mol Sci. 2020 Mar 4;21(5):1747. doi: 10.3390/ijms21051747 (PMC7084392; doi:10.3390/ijms21051747)
Supplement: Supplementary file 1 [file ijms-21-01747-s001.pdf]

Table S1: shRNA sequences

Mouse shRNA	Sequence
shFoxA2-A	5′-GCACAAGCGAGGTGGCCTA-3′
shFoxA2-B	5′-TGAATGGCATGAACACATA-3′

Table S2: qPCR primer sequences of mouse genes.

Gene(mouse)	Forward (5′ to 3′)	Reverse (5′ to 3′)
**Tnfα**	CCCTCACACTCAGATCATCTTC	GCTACGACGTGGGCTACAG
**Il6**	TCTATACCACTTCACAAGTCGGA	GAATTGCCATTGCACAACTCTTT
**Cxcl10**	CCAAGTGCTGCCGTCATTTTC	GGCTCGCAGGGATGATTTCAA
**Mcp1**	TTAAAAACCTGGATCGGAACCAA	GCATTAGCTTCAGATTTACGGGT
**Foxa2**	GAGCACCATTACGCCTTCAAC	AGGCCTTGAGGTCCATTTTGT
**Atrogin**	TGTGGGTGTATCGGATGGAGA	CTGCATGATGTTCAGTTGTAAG
**MuRF1**	ACGAGAAGAAGAGCGAGCTG	CTTGGCACTTGAGAGGAAGG
**Mstn**	TGACGATTATCACGCTACCACG	GTAGGAGTCTTGACGGGTCTG

Table S3: ChIP primer sequence

ChIP Primers	Forward (5′ to 3′)	Reverse (5′ to 3′)
**FoxA2 PPRE1**	CTTTGGGCACCTTGGATTTA	AAGCAGGTGACAGCCAATTT
**FoxA2 PPRE2**	CCAGAGGACTTGGTGTTGGT	TCATGTTGCTCACGGAAGAG
**Negative PPRE Control**	GGAGAGTGGGCAATGGAATA	TGCTTAACGCTGTGCAATTC

Table S4: Nucleotide sequences of promoter region for luciferase reporter constructs

**Insert**	**Gene product sequence (5′ - 3′)**
**PPRE1**	cggggtaccccacctactgccctgtttgttttagttacgaaatgctttgggcaccttggatttaactgaaaagtaaccttgaaa<br>caccgaggccctcatgccagaggcaaatcgctgcctcccgggtattggctgcagctaaacgggtctctccaggccgactg<br>aggtgggtagccagaaagaggactgaggtaactgacgaccagggcggccagaccacgcgagtcctacgcgcctcctg<br>aggccgccccgggacttaactgtaacggggaggggcctccggagcagcggccagcgagttaaaggtgtgtacacagtt<br>tttctaaataggacagcgctttgcaaattggctgtcacctgcttgttgttttgacagggaatgtgcactaaaagggaggaaa<br>cccgagataaataaatctggcttgcctcgctctcgagcgg
**ΔPPRE1**	cggggtaccccacctactgccctgtttgttttagttacgaaatgctttgggcaccttggatttaactgaaaagtaacctgaa<br>acaccgaggccctcatgccagaggcaaatcgctgcctcccgggtattggctgcagctaaacgggtctctccaggccgac<br>tgaggtgggtagccagaaagag**tc**ctgaggtaactgacgaccagggcggccagaccacgcgagtcctacgcgcctcc<br>tgaggccgccccgggacttaactgtaacggggaggggcctccggagcagcggccagcgagttaaaggtgtgtacac<br>agtttttctaaataggacagcgctttgcaaattggctgtcacctgcttgttgttttgacagggaatgtgcactaaaagggag<br>gaaacccgagataaataaatctggcttgcctcgctctcgagcgg

Mutated sequences are indicated in red.
The promoter region harboring the PPRE was inserted between the Kpn1 and XhoI sites of pGL2-promoter vector (Promega, USA)

Supplemental Figures

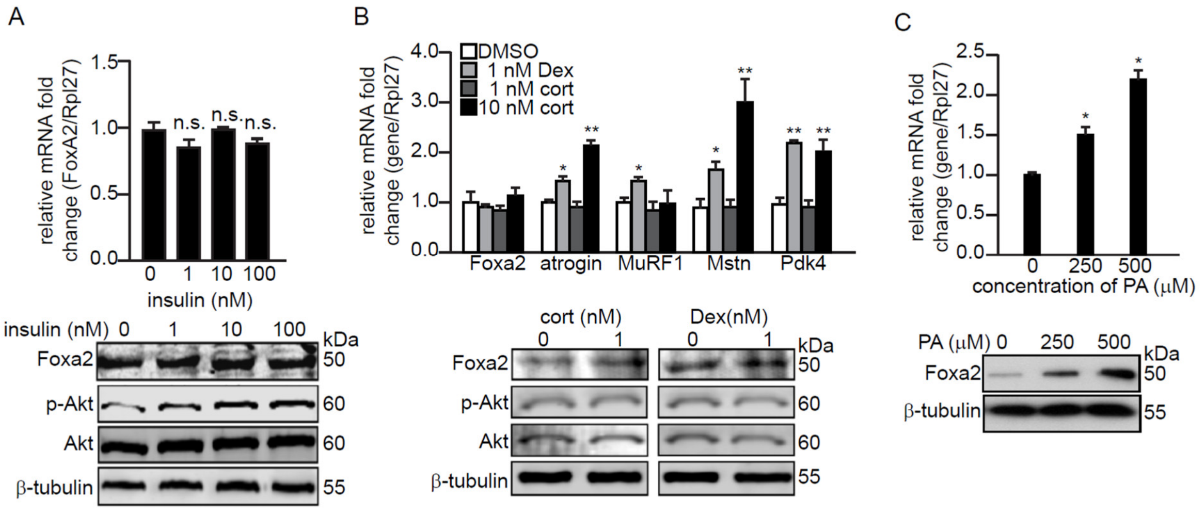

Figure S1. FoxA2 expression in C2C12 cells is upregulated by palmitate fatty acid. **(A,C)** Relative FoxA2 mRNA and protein expression in C2C12 cells treated with various concentration of insulin (**A**) and palmitate fatty acid (PA) for 24 h (**C**). Values are the mean+s.d. from 3 independent experiments. *p< 0.05; **p< 0.01, n.s., not significant. **(B)** Relative fold change in mRNA levels of FoxA2, Atrogin, MuRF1 and myostatin (Mstn) in C2C12 cells treated with indicated concentration of hydrocortisone (cort) or dexamethasone (Dex). The increased expression of phosphorylated Akt and Pdk4 mRNA serve as positive controls for insulin treatment and Dex and cort, respectively. Values are the mean+s.d. from 3 independent experiments. * $p < 0.05$; ** $p < 0.01$.

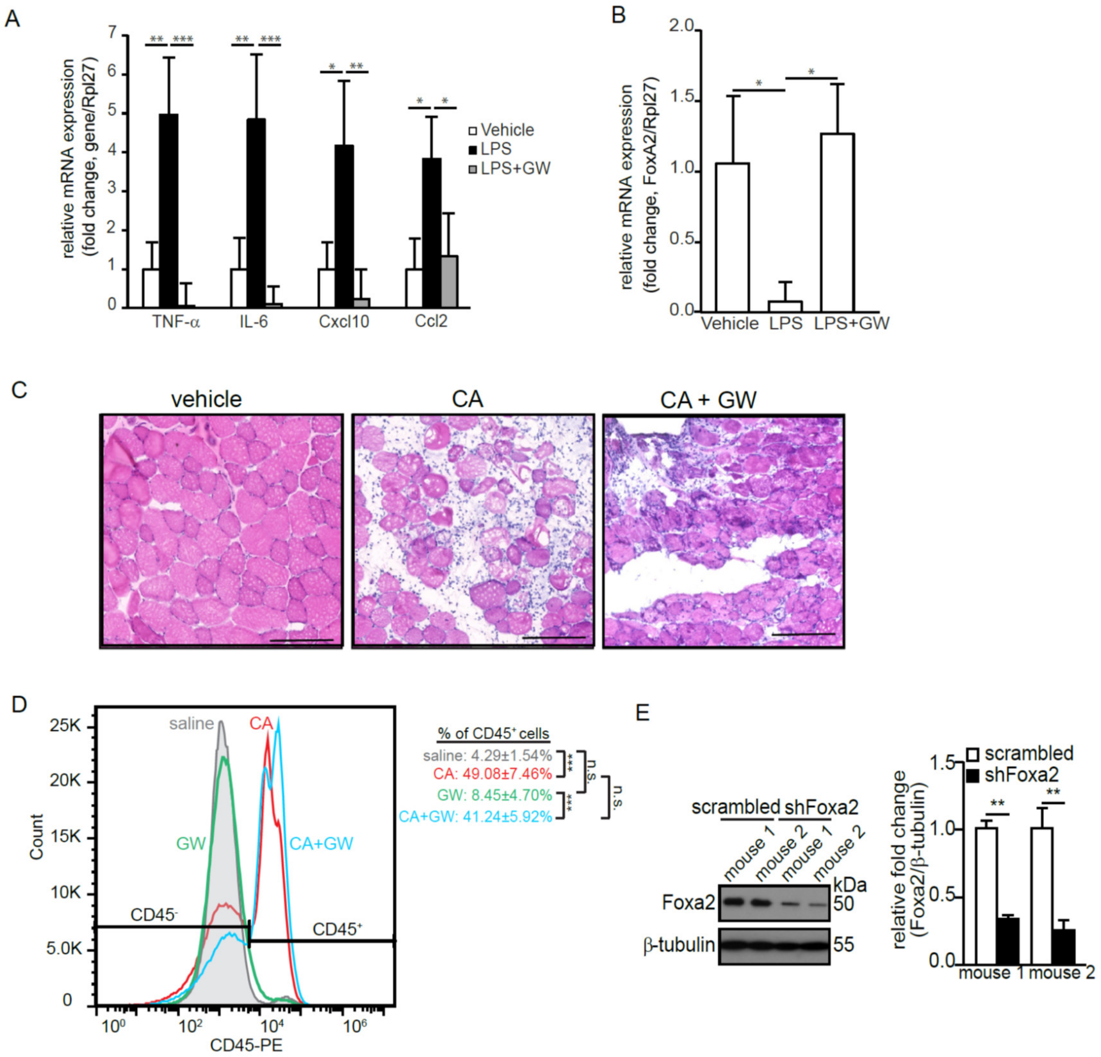

Figure S2. PPARβ/δ agonist treatment reduces pro-inflammatory cytokines in CA-induced muscle inflammation. (**A**,**B**) Relative mRNA levels of proinflammatory genes (**A**), and FoxA2 (**B**) in gastrocnemius muscle at 24 h post LPS-induced inflammation with or without GW501516 (GW) pretreatment. Values are the mean ± s.d. from n= 5 mice per group. *p< 0.05; **p< 0.01, ***p<0.001. (**C**) Representative cross-sectional hematoxylin and eosin (H&E) stains of TA muscles at 6 h of CA-induced inflammation (CA only) and in the presence of GW co-treatment (CA+GW), compared to vehicle(saline)-treated mice. Scale bar: 100 µm. **(D)** Representative histogram plots of infiltrated CD45-positive (CD45+) immune cells from TA muscle from wild-type and PPARβ/δ-deficient mice subjected to indicated treatments. Three mice per experiments were used for each treatment. n=3 independent studies. ** $p < 0.01$, ***$p < 0.001$, n.s., not significant. **(E)** Relative FoxA2 protein expression in TA muscle injected with either ad-shFoxA2 or Ad-scrambled on collateral hindlimb. Representative immunoblot of FoxA2 is shown. β-tubulin serves as a loading control from the same samples. Five mice are used for each treatment. **p<0.01.
